# Effects of intermittent pneumatic compression devices interventions to prevent deep vein thrombosis in surgical patients: A systematic review and meta-analysis of randomized controlled trials

**DOI:** 10.1371/journal.pone.0307602

**Published:** 2024-07-23

**Authors:** Nam Young Kim, Seang Ryu, Yun-Hee Kim

**Affiliations:** 1 Department of Nursing, Jungwon University, Goesan, Korea; 2 Department of Nursing, Mokpo National University, Muan, Korea; University Magna Graecia of Catanzaro, ITALY

## Abstract

This review aimed to determine the effectiveness of Intermittent Pneumatic Compression (IPC) intervention on Deep Vein Thrombosis (DVT) in surgical patients. An electronic database search was conducted with PubMed, OVID-MEDLINE, EMBASE, and CENTRAL, from September 22 to 28, 2023. Three researchers independently selected the studies, assessed their methodological quality, and extracted relevant data. We conducted a meta-analysis of the effect of IPC versus the control group and summarized the intervention results from the included studies. Of the 2,696 articles identified 16 randomized control trials met the inclusion criteria for review. IPC interventions significantly affected DVT prevention (OR = 0.81, 95% CI: 0.59–1.11). In the subgroup analysis, there was a significant pooled effect (OR = 0.41, 95% CI: 0.26–0.65]), when the comparison group was no prophylaxis group. However, when the comparison groups were the pharmacologic prophylaxis group ([OR = 1.32, 95% CI 0.78–2.21]) and IPC combined with the pharmacologic prophylaxis group (OR = 2.43, 95% CI: 0.99–5.96) did not affect DVT prevention. The pooled effects of Pulmonary Embolism (PE) (OR = 5.81, 95% CI: 1.25–26.91) were significant. IPC intervention showed a significant effect on bleeding prevention (OR = 0.17, 95% CI: 0.08–0.36) when compared to IPC combined with the pharmacologic groups. IPC intervention effectively prevented DVT, PE, and bleeding in surgical patients. Therefore, we propose that IPC intervention be applied to surgical patients to avoid DVT, pulmonary embolism, and bleeding in the surgical nursing field as scientific evidence suggests.

## Introduction

Deep vein thrombosis (DVT), along with pulmonary embolism (PE), is a dangerous condition that can have devastating consequences for patients and occurs in the deep veins following venous injury due to prolonged immobilization, paralysis, surgery, or trauma [1, 2]. Hospitalization for surgery has been consistently identified as one of the most critical risk factors for DVT, with the incidence of DVT after surgery reported to be 10–14% [3]. Patients who develop DVT have prolonged hospital stays, increased medical costs, and may even experience disability or death [4]. However, it has been reported that DVT prophylaxis can reduce the incidence of DVT by as much as 2% when applied appropriately to patients [3]. It is essential to prevent DVT rather than treat it after it occurs and provide appropriate prevention during inpatient care for surgical patients, who constitute a significant risk group for DVT [5, 6].

Various prophylactic measures can be applied to patients at risk of developing DVT. In surgical patients, the main therapies are non-pharmacological (graduated compression stockings (GCS), intermittent pneumatic compression (IPC), and pharmacological prophylaxis) [5, 7, 8]. The mechanism of DVT is still unclear, but it is known to be caused by damage to the inner wall of the blood vessel, stagnation of blood flow, and hypercoagulation [9, 10]. In particular, in surgical patients, muscle weakness and decreased blood flow due to immobilization have been identified in previous studies as major factors affecting the occurrence of DVT [11]. GCS and IPC are non-pharmacological therapies that reduce venous blood pooling in the lower extremities and increase blood flow velocity; they have fewer side effects than pharmacological prophylaxis but are often used as an adjunct to pharmacological prophylaxis in clinical practice [12].

The Scottish Intercollegiate Guidelines Network (SIGN) [7] guidelines for DVT prophylaxis recommend IPC as the primary treatment for DVT prophylaxis in patients undergoing general surgery or cardiac surgery and IPC in combination with pharmacological prophylaxis in patients undergoing total hip or total knee replacement surgery when there is no risk of bleeding.

However, the 9th American College of Chest Physicians Antithrombotic Therapy and Prevention of Thrombosis Guidelines recommend the use of low-molecular-weight heparin (LMWH) to treat blood clots and prevent thrombosis [13]. A meta-analysis of randomized controlled trials (RCTs) in critically ill patients concluded that the evidence for the effectiveness of IPC compared to pharmacological prophylaxis is not clear [14], suggesting conflicting views on the effectiveness of IPC.

Since 2010, studies evaluating the effects of IPC in surgical patients have found that IPC reduces the incidence of DVT. However, some studies have reported that IPC did not affect the incidence of DVT compared to the control group [A1-A3, A5, A9-A12, A15, A16 in S1 Appendix]. Despite the widespread use of IPC in conjunction with pharmacologic prophylaxis [15], it is difficult to find clear evidence of the effect of IPC on the incidence of DVT in surgical patients.

Most previous meta-analyses have shown that the effect of IPC on DVT incidence of DVT have focused on critically ill patients. In 2020, a meta-analysis of ten studies on the prevention of DVT in critically ill patients showed that the IPC group had a lower incidence of DVT than the no-prophylaxis group. However, there was no significant difference in the incidence of Venous Thromboembolism (VTE) between the IPC and LMWH groups, and the IPC group reported less bleeding than the LMWH group [8]. Therefore, it is necessary to analyze whether there is a difference in the incidence of DVT and PE in surgical patients compared with other interventions.

Furthermore, it is necessary to systematically review the application of IPC in surgical patients and suggest specific measures to make it more convenient for clinical nurses to implement IPC interventions. Therefore, this study aimed to analyze the effectiveness of IPC in preventing DVT in surgical patients.

## Materials and methods

### Eligibility criteria

This study was conducted according to the Cochrane Handbook for Systematic Reviews of Interventions [16] and written according to the PRISMA reporting guidelines [17].

Study participants were selected according to the PICOS-SD (Participants, Intervention, Comparison, Outcome, Setting, and Study Design). (1) Participants: adult patients aged 19 years or older hospitalized in a surgical ward after undergoing IPC. (2) Intervention: IPC. (3) Comparison of thromboprophylaxis, combined thromboprophylaxis and IPC, and no intervention. (4) Outcomes: Incidence of DVT, PE, and bleeding after IPC. (5) Setting: All surgical wards. (6) Study design: RCTs. Studies that did not include surgical patients and those that were not RCTs were excluded.

### Systematic search

A systematic search was conducted from September 22 to 28, 2023. The selection of search databases was based on the COre, Standard, Ideal model proposed by the U.S. National Library of Medicine [18], Ovid, EMbase, and CENTRAL were used for core databases, and CINAHL was used for standard databases. For the systematic search, we used Medical Subject Headings (MeSH) and the Index of Life Sciences Terms (EMTREE) and utilized search functions such as Boolean operators and truncations.

For the participants, we used “postoperative care” [MeSH], “postoperative period” [MeSH], and Surgical OR Surgery [text words]. The search term of intervention was used as “Intermittent pneumatic compression devices” [MeSH], “pneumatic NEAR compression*” OR “compression NEAR device* OR sequential NEAR compression*” [Textword], “flowtron OR IPC” [Textword], “intermittent pneumatic compression device” OR “A-V Impulse System” OR “ArtAssist” OR “Flexitouch system” OR “FLOWTRON” OR “intermittent pneumatic compression devices” OR “Plexipulse” OR “pneumatic intermittent impulse device” OR “SC-2004 Sequential Circulator PCD” OR “Walkcare” OR “assisted circulation” OR “bandage” OR “compression instrument” OR “compression device” OR “intermittent compression” OR “intermittent pneumatic” OR “foot pump” OR “foot-pumps” OR “foot-pump” OR”compression garm” [Textword]. The RCT search filter of the SIGN was used to identify randomized trials. There were no restrictions on the year of publication or language.

### Data selection and extraction

The retrieved data were managed using a bibliographic program (EndNote 20). The literature was first screened by removing duplicate articles and reviewing the titles and abstracts. Based on the inclusion and exclusion criteria, the articles were screened twice and finally selected for inclusion in this systematic review. The literature review was conducted independently by three researchers, and in case of disagreement, a consensus was reached through discussion based on the inclusion and exclusion criteria.

Three researchers independently collected the studies for the systematic review according to the data extraction form designed by the research team. The data included the author’s name, year, country of publication, number of subjects, age, sex, body mass index (BMI), type of intervention (IPC type, method of application, and duration of intervention), and control group. The outcome variables included DVT, PE, and bleeding incidence.

### Assessment of methodological quality

The reviewers used the Cochrane Collaboration’s RCT quality assessment tool, Risk of Bias (RoB) 2.0 [19]. The tool comprises the following signal questions: “randomization process,” “deviations from the intended interventions,” “missing-outcome data,” “measurement of the outcome,” and “selection of the reported result.” The signal questions were evaluated and responded with yes, probably yes, probably no, or no. Based on the signal question responses in each domain, the risk of bias was determined as low, some concern, or high according to the assessment algorithm presented in RoB 2. Finally, the risk of bias was assessed as low, some concern, or high based on the overall literature quality assessment criteria presented in RoB 2.0 [19]. The final selection process was conducted independently by three researchers by obtaining the original full texts, and in case of disagreement, a consensus was reached through discussion.

### Data analysis

The preventive effect of IPC on DVT, PE, and bleeding incidence was estimated by meta-analysis using the Cochrane Collaboration’s Review Manager 5.4. The number of DVT, PE, and bleeding events was calculated from the total number of patients in the experimental and control groups, and the pooled effect size estimate was calculated as the odds ratio (OR). The overall effect size was divided into subgroups: no prophylaxis, pharmacological thromboprophylaxis, and IPC plus pharmacological thromboprophylaxis.

The chi-square test and Higgin’s I^2^ test were used to determine heterogeneity, with Higgin’s I^2^ = 0% indicating no heterogeneity, 25% indicating small-sized heterogeneity, 50% indicating moderate heterogeneity, and 75% or more indicating considerable heterogeneity [16]. The random-effects model was used for analysis when I^2^ = 75% or higher, and the fixed-effects model was used when I^2^ = 75% or lower.

## Results

### Data selection

**Fig 1:**
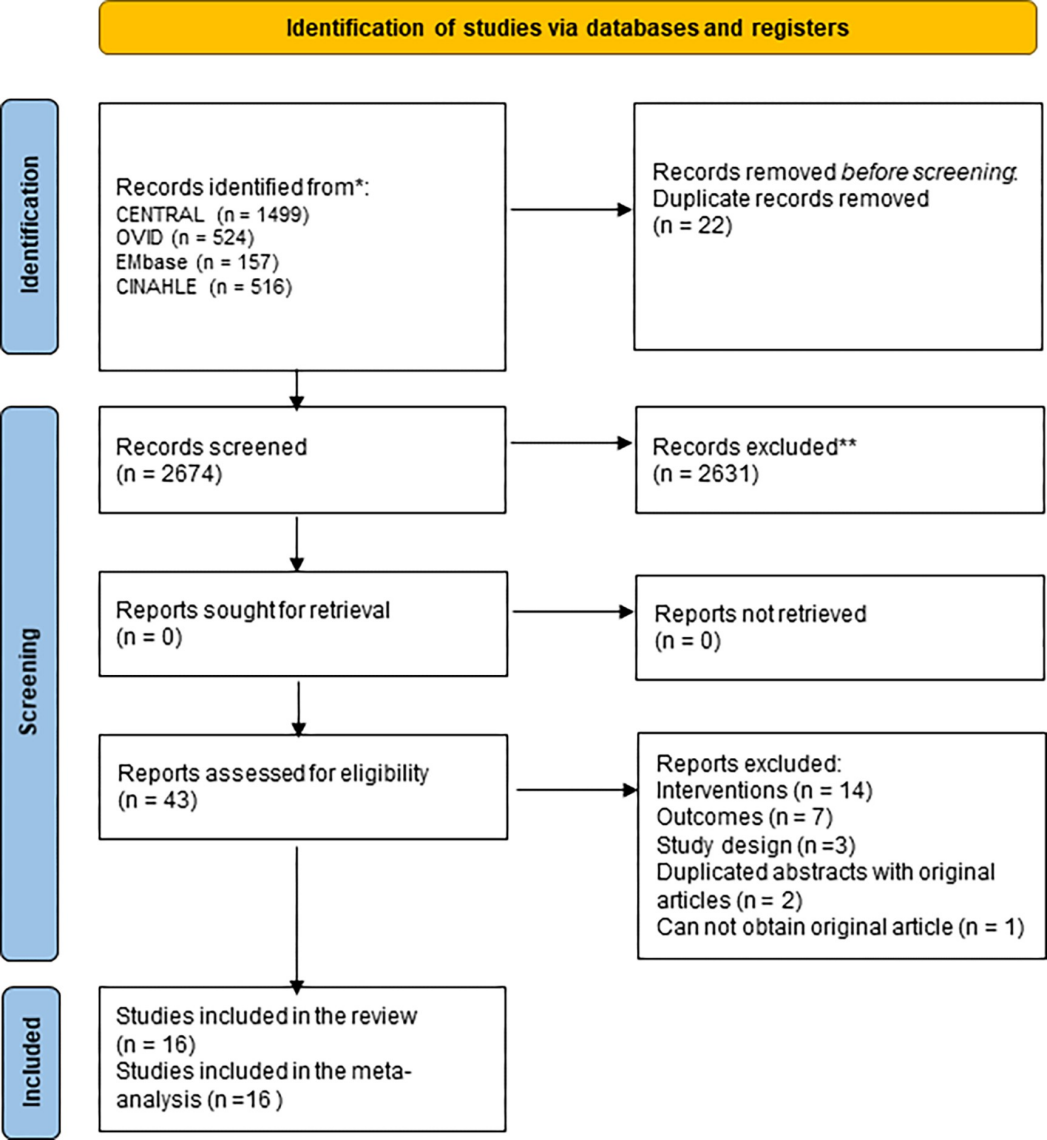
PRISMA flow of study selection process.

The systematic search yielded 1499 articles in CENTRAL, 524 in OVID, 157 in EMbase, and 516 in CINAHL. A primary search was conducted using EndNote 20 to remove 22 duplicate articles. After eliminating duplicates, three researchers reviewed the titles and abstracts and excluded 2631 out of 2674 articles. The remaining 43 articles were obtained and reviewed according to the inclusion and exclusion criteria. Seven studies did not present outcome variables, 14 were not interventions, and three were not RCTs. Further, two abstracts published in duplicate and one study not available through the overseas data request service of a Korean library were excluded. Finally, 16 studies were included in the systematic review (Fig 1).

### Assessment of methodological quality

As a result of evaluating the risk of bias in the five areas of the quality assessment tool according to the evaluation algorithm, six studies were rated as “low” for the risk of bias in the randomization process. A total of 10 studies were rated as “low” for the risk of bias due to deviation from the intended intervention. Further, 16 studies were rated as “low” for the risk of bias due to missing intervention outcome data, and 13 were rated as “low” for the risk of bias in measuring the intervention outcome. Based on the above results, the overall quality assessment of studies was analyzed according to the assessment algorithm presented in RoB 2.0. Moreover, four studies were rated as having a “low” risk of bias. One study was rated as having “some concern,” and 11 studies were rated as having a “high” risk of bias (Table 1).

**Table 1:** Descriptive summary of trials and risk of bias summary (N = 16).

No	Author (year) Country	Sample size N	Age (year) M(SD)	Sex, Male N	BMI(kg/m^2^) M(SD)	Diagnostic criteria for DVT	Types of surgery	Risk of bias summary					
								R	D	M 1	M2	S	O
A1	Domeij-Arverud et al. (2013) Sweden	E: 14	E: 39.8(NI)	E: 12	E: 27.2(NI)	Ultrasonography	Repair of tendo achillis rupture	L	SC	L	L	L	SC
		C: 12	C: 40.4(NI)	C: 10	C: 24.3(NI)								
A2	Domeij-Arverud et al. (2015) Sweden	E: 69	E: 40.9(NI)	E: 61	E: 27.1(NI)	Ultrasonography	Repair of tendo achillis rupture	L	L	L	L	L	L
		C: 71	C: 39.9(NI)	C: 58	C: 26.7(NI)								
A3	Coe et al. (1978) USA	E: 29	E: 55(11)	E: NI	E: NI	Fibrinogen scan phlebograms	Urological surgery	H	L	L	L	L	H
		C1: 24	C1: 51(18)	C1: NI	C1: NI								
		C2: 28	C2: 63(16)	C2: NI	C2: NI								
A4	Butson (1981) Canada	E: 62	E: 57.5(NI)	E: 26	E: NI	Fibrinogen scan	Abdominal surgery	H	L	L	L	S	H
		C: 57	C: 52.4(NI)	C: 26	C: NI								
A5	Skillman et al. (1978) USA	E: 47	E: 49.0(NI)	E: NI	E: NI	Fibrinogen scan phlebograms	Neuro surgery	H	L	L	L	L	H
		C: 48	C: 48.7(NI)	C: NI	C: NI								
A6	Wilson et al. (1992) UK	E: 28	E: 71.1(6.7)	E: 5	E: NI	Ultrasonography	Total knee replacement	H	L	L	L	SC	H
		C: 31	C: 70.1(6.8)	C: 10	C: NI								
A7	Wang et al. (2013) China†	E: 60	E: NI	E: NI	E: NI	Ultrasonography	Retal cancer resection	SC	H	L	L	SC	H
		C: 60	C: NI	C: NI	C: NI								
A8	Blanchard et al. (1999) Switzerland	E: 63	E: 72(NI)	E: 11	E: 44.7(NI)	Phlebograms	Total knee replacement	H	L	L	L	L	H
		C: 67	C: 74(NI)	C: 20	C: 43.6(NI)								
A9	Clarke-Pearson et al. (1993) USA	E: 101	E: 55(NI)	E: NI	E: NI	Fibrinogen scan	Gynecologic oncology surgery	L	L	L	L	L	L
		C: 107	C: 57(NI)	C: NI	C: NI								
A 10	Maxwell et al. (2001) USA	E: 106	E: 62(NI)	E: NI	E: NI	Ultrasonography	Gynecologic oncology surgery	L	H	L	L	L	H
		C: 105	C: 60(NI)	C: NI	C: NI								
A 11	Nagata et al. (2015) Japan	E: 14	E: 53.2(10.9)	E: NI	E: 21.2(2.0)	Ultrasonography	Gynecologic oncology surgery	L	L	L	L	L	L
		C: 16	C: 60.5(10.7)	C: NI	C: 23.394.6)								
A 12	Pitto et al. (2004) New Zealand	E: 100	E: 57.3(12)	E: 30	E: 27.8(3.2)	Ultrasonography	Total hip replacement	H	H	L	L	L	H
		C: 100	C: 58.1(11)	C: 32	C: 28.1(2.9)								
A 13	Stannard et al. (1996) USA	E: 25	E: 68.7(NI)	E: NI	E: NI	Ultrasonography	Total hip arthroplasty	L	L	L	L	L	L
		C1: 25	C1: 69.7(NI)	C1: NI	C1: NI								
		C2: 25	C2: 65(NI)	C2: NI	C2: NI								
A 14	Jung et al. (2018) Korea	E: 336	E: 57.4(10.3)	E: 230	E: 23.7(2.7)	Ultrasonography	Gastrectomy	H	SC	L	L	L	H
		C: 330	C: 57.9(10.9)	C: 205	C: 23.9(3.0)								
A 15	Song et al. (2014) Korea	E: 112	E: 58.8(9.7)	E: 83	E: 23.8(2.5)	Ultrasonography	Gastrectomy	H	L	L	L	L	H
		C: 108	C: 56.3(11.1)	C: 67	C: 23.7(2.8)								
A 16	Kamachi et al. (2020) Japan	E: 223	E: 65.0(9.3)	E: 125	E: 23.3(3.3)	Multidetector CT	Laparoscopic surgery for gastric cancer	H	SC	L	L	L	H
		C: 225	C: 64.8(9.1)	C: 132	C: 23.7(3.7)								

BMI = body mass index; C = control group; D = deviations from intended intervention; DVT = deep vein thrombosis; E = experimental group; H = high risk of bias; L = low risk of bias; M = mean; M1 = missing outcome data; M2 = measurement of the outcomes; NI = no information; O = overall bias; R = randomization process; S = selection of the reported; SC = some concern; SD = standard deviation; †Abstract

### Characteristics of the included studies

The general characteristics of the 16 included studies are shown in Table 1. The total number of participants was 2,828 (1,389 in the intervention group and 1,436 in the control group), with the United States being the most common country of origin (5 studies). The average age of participants ranged from 39.8 to 74 years. The most common method used to diagnose DVT was ultrasonography, which was reported in 10 studies (Table 1).

Next, when looking at the IPC characteristics provided to the experimental group, a knee-length application was the most common (seven studies), and intermittent IPC application mode was the most common (nine studies). Regarding the timing of IPC administration, six studies applied it at the start of anesthesia, five applied it postoperatively, and four applied it preoperatively. Finally, all 16 studies reported the incidence of DVT as an effect of IPC. Six studies reported the incidence of PE, and four studies reported the incidence of bleeding as an effect of IPC (Table 2).

**Table 2:** Details of interventions and outcomes (N = 16).

Author (year)	Sample size(n)		Interventions (IPC)				Comparisons	Primary Outcomes (No, of events)				Secondary Outcomes (No, of events)	
	E	C	IPC sleeve type	Apply Mode of IPC	Initiation	Termination		DVT		PE		Bleeding	
								E	C	E	C	E	C
Domeij-Arverud et al. (2013)	14	12	Foot-length	Non-sequential	Post-OP	Trial day 14	No prophylaxis	^6^	5	-^‡^	-^‡^	-^‡^	-^‡^
Domeij-Arverud et al. (2015)	69	71	Knee-length	Intermittent	Post-OP	Trial day 14	No prophylaxis	16	26	-^‡^	-^‡^	-^‡^	-^‡^
Coe et al. (1978)	29	24	Knee-length	Intermittent	Induction of anesthesia	Hospital discharge	No prophylaxis	2	6	-^‡^	-^‡^	-^‡^	-^‡^
		28					Heparin		6				
Butson et al. (1981)	62	57	Knee-length	Non-sequential	Induction of anesthesia	24–48 hours	No prophylaxis	6^†^	4	-^‡^	-^‡^	-^‡^	-^‡^
Skillman et al. (1978)	47	48	Knee-length	Intermittent	Induction of anesthesia	Until bed rest after surgery	No prophylaxis	4	12	-^‡^	-^‡^	-^‡^	-^‡^
Wilson et al. (1992)	28	31	Limb-length	Continuously	Post-OP	Trial day 9 or 10	No prophylaxis	5^†^	19	-^‡^	-^‡^	-^‡^	-^‡^
Wang et al. (2013)	60	60	NI	NI	NI	NI	No prophylaxis	1^†^	8	-^‡^	-^‡^	-^‡^	-^‡^
Blanchard et al. (1999)	63	67	Knee-length	Continously	Pre-OP	Hospital discharge	LMWH	34^†^	16	-^‡^	-^‡^	-^‡^	-^‡^
Clarke-Pearson et al. (1993)	101	107	Calf-length	NI	Induction of anesthesia	Trial day 5 or Hospital discharge	Heparin	4	7	-^‡^	-^‡^	-^‡^	-^‡^
Maxwell et al. (2001)	106	105	Knee-length	Intermittent	Induction of anesthesia	Trial day 5	LMWH	1	2	0	0	3	4
Nagata et al. (2015)	14	16	Below-knee	Continuously	Pre-OP	Walking independently	LMWH	3	1	3^†^	0	-^‡^	-^‡^
Pitto et al. (2004)	100	100	Foot-length	Intermittent	Post-OP	Anytime (when the pt wants)	LMWH	3	6	0	0	-^‡^	-^‡^
Stannard et al. (1996)	25	25	Foot-length	Intermittent	Post-OP	Hospital discharge	Heparin+aspirin	0^†^	5	-^‡^	-^‡^	-^‡^	-^‡^
		25					IPC+heparin+aspirin	0	0	-^‡^	-^‡^	-^‡^	-^‡^
Jung et al. (2018)	336	330	Limb-length	Intermittent	Pre-OP	Hospital discharge	IPC+LMWH	11^†^	2	1	0	4^†^	30
Song et al. (2014)	112	108	NI	Intermittent	Pre-OP	Hospital discharge	IPC+LMWH	3	0	0	0	1	11
Kamachi et al. (2020)	223	225	Knee-length	Intermittent	Induction of anesthesia	POD 1 day	IPC+LMWH	6	5	6	1	0	11

C = control group; DVT = deep vein thrombosis; E = experimental group; IPC = intermittent pneumatic compression; LMWH = low molecular-weight heparin; NI = no information; OP = operation; PE = pulmonary embolism; POD = Postoperative Day

†p<.05

‡Not reported

### Effects of intermittent pneumatic compression intervention in surgical patients

#### Effects on deep vein thrombosis prevention

**Fig 2:**
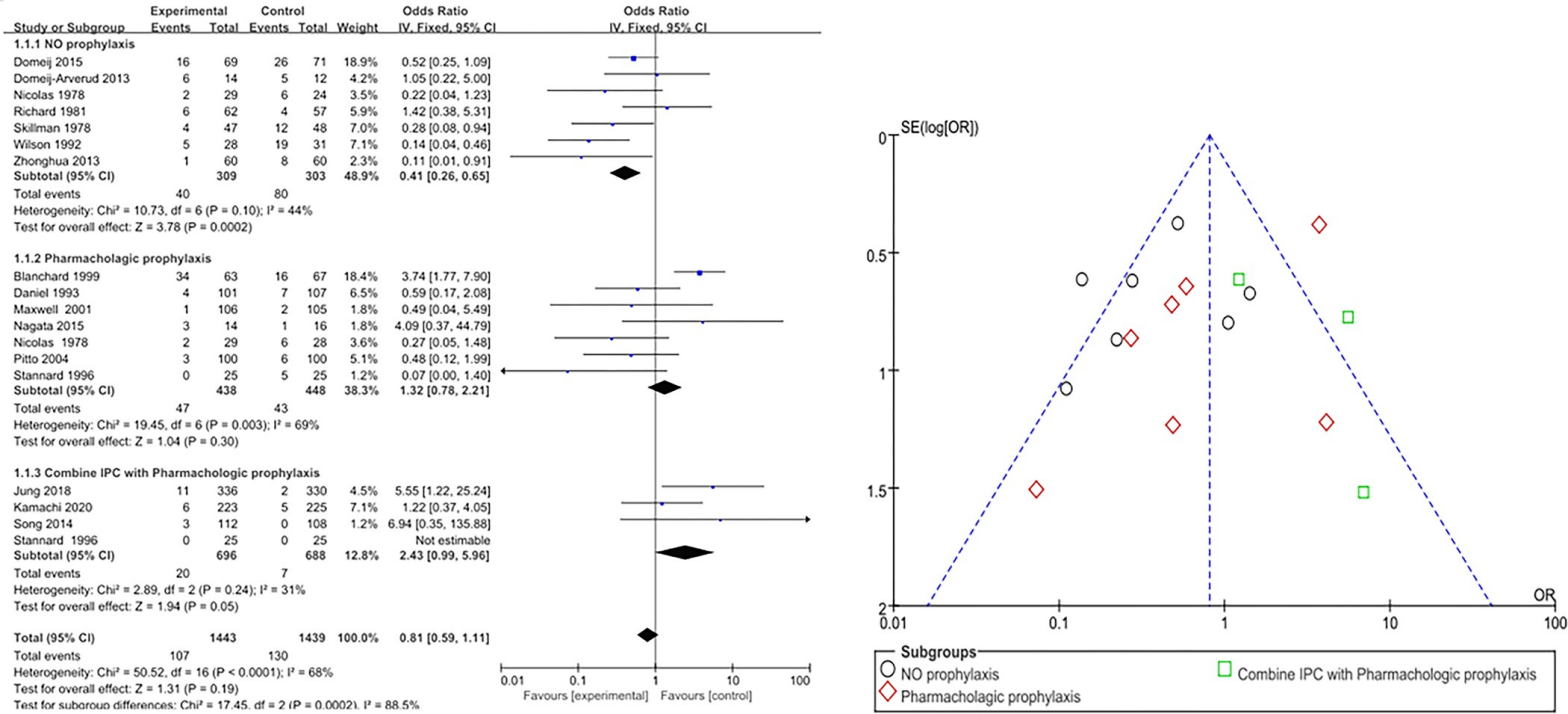
Effects of IPC on deep vein thrombosis prevention.

The overall effect size of IPC for DVT prevention was OR 0.81 (95% CI: 0.59–1.11), which was not statistically significant (Z = 1.31, *p* = .190). Heterogeneity was I^2^ = 68% (χ ^2^ = 50.52, df = 16, *p*< .001). Subgroup analysis showed a statistically significant difference in DVT prevention effect size with an OR of 0.41 (95% CI: 0.26–0.65) when the comparison group was the no prophylaxis group (Z = 3.78, *p* < .001). Heterogeneity was I^2^ = 44% (χ^2^ = 10.73, df = 6, *p* = .100). When the comparison arm was pharmacologic prophylaxis, the effect size for DVT prevention was OR of 1.32 (95% CI: 0.78–2.21), which was not statistically significant (Z = 1.04, *p* = .300). Heterogeneity was I^2^ = 69% (χ^2^ = 19.45, df = 6, *p* = .003). When the comparison group was IPC plus pharmacologic prophylaxis, the effect size for DVT prevention was OR of 2.43 (95% CI: 0.99–5.96), which was not statistically significant (Z = 1.94 *p* = .050). The heterogeneity was I^2^ = 31% (χ^2^ = 2.89, df = 2, *p* = .240) (Fig 2).

#### Effects on pulmonary embolism prevention

**Fig 3:**
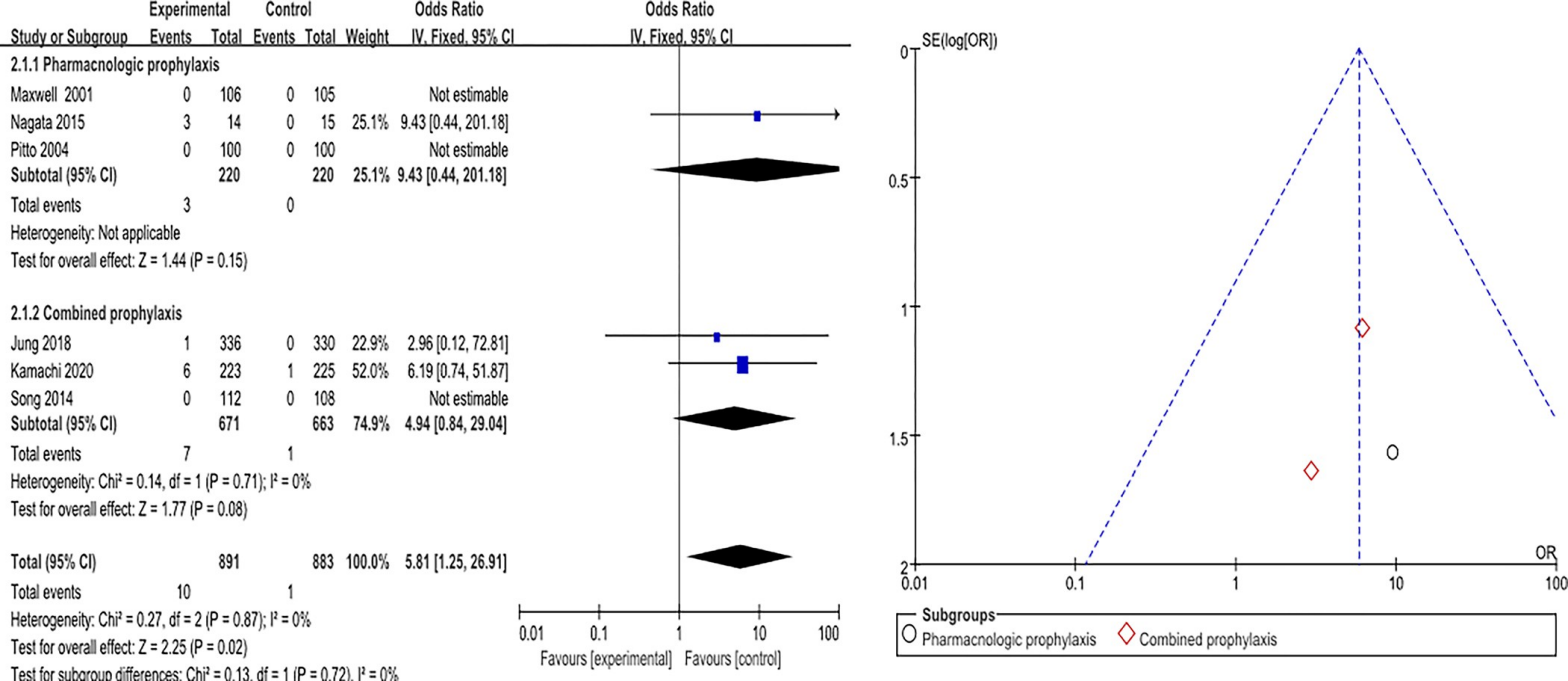
Effects of IPC on pulmonary embolism prevention.

The overall effect size of IPC for PE prevention was OR of 5.81 (95% CI: 1.25–26.91), which was statistically significant (Z = 2.25, *p* = .020). Heterogeneity was I^2^ = 0% (χ^2^ = 0.27, df = 2, *p* = .870). Subgroup analysis showed that the effect size for PE prevention was not statistically significant when the comparator was pharmacologic prophylaxis, with an OR of 9.43 (95% CI: 0.44–201.18) (Z = 1.44, *p* = .150). Heterogeneity was not assessed in this study. When the comparison arm was a combination of IPC and pharmacologic prophylaxis, the effect size for PE prevention was OR of 4.94 (95% CI: 0.84–29.04), which was not statistically significant (Z = 1.77, *p* = .080). I^2^ = 0.0% (χ^2^ = 0.14, df = 1, *p* = .710) (Fig 3).

#### Effects on bleeding prevention

**Fig 4:**
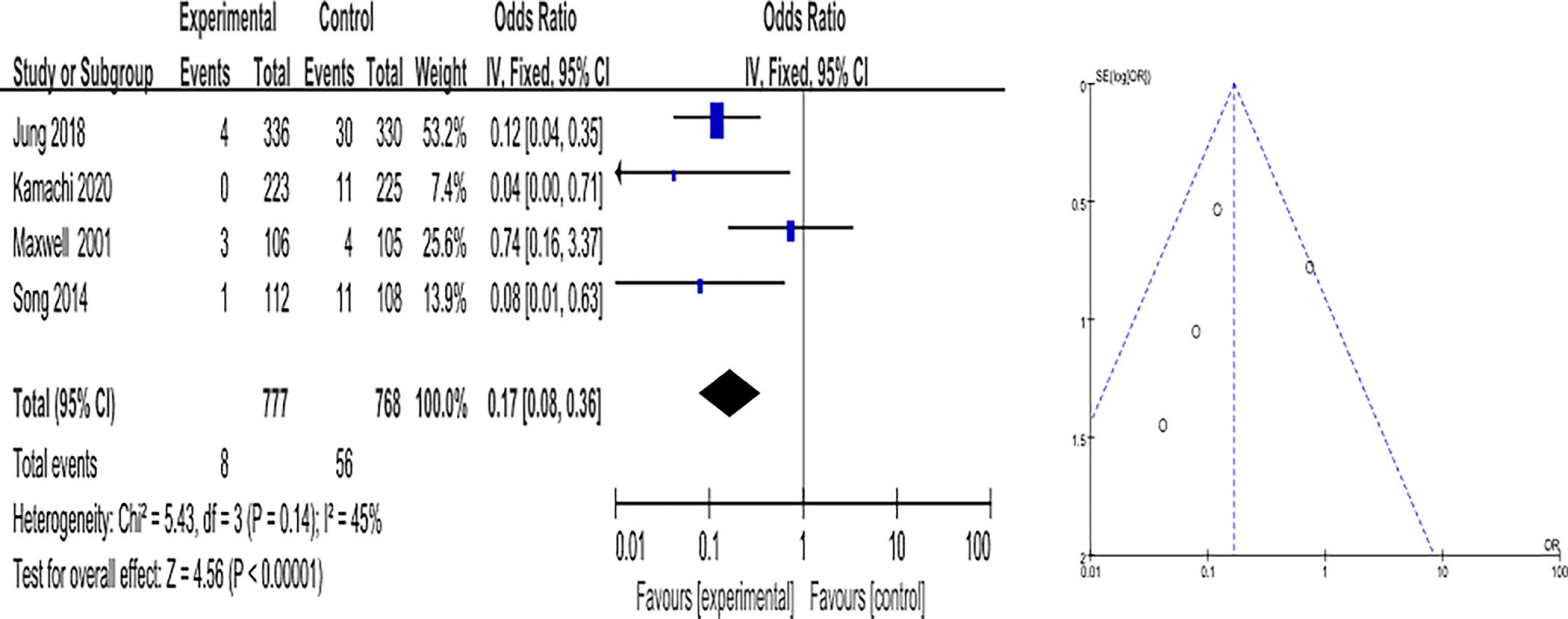
Effects of IPC on bleeding.

The overall effect size for bleeding prevention of IPC was OR of 0.17 (95% CI: 0.08–0.36), which was statistically significant (Z = 4.56, *p* < .001) compared to anticoagulation. Heterogeneity was I^2^ = 45% (χ^2^ = 5.43, df = 3, *p* = .140) (Fig 4).

### Publication bias

Publication bias for the selected data was visually judged using funnel plots. The effects of IPC interventions on DVT, PE, and bleeding were symmetrical, indicating a low risk of publication bias (Figs 2–4).

## Discussion

We conducted a systematic review of RCTs and a meta-analysis of outcome variables to determine the effect of postoperative IPC on the incidence of DVT, PE, and bleeding in patients hospitalized in surgical wards.

The effects of the IPC identified in this study were compared with those of previous meta-analysis studies. All 16 RCTs included in this study reported the DVT incidence, and a meta-analysis of the overall effect size of DVT incidence across the 16 studies showed no difference in DVT incidence between the experimental and control groups. However, because the DVT prophylaxis applied to the control group in the 16 studies differed, the subgroups were analyzed as the no prophylaxis group, pharmacological prophylaxis group, and combination of pharmacological prophylaxis and IPC. Of the three subgroups, only the no-prophylaxis group had a significantly higher incidence of DVT than the experimental group. In a systematic review and meta-analysis of nine studies in critically ill patients, the incidence of DVT in the no-prophylaxis group was higher than that in the experimental group with IPC but not in the pharmacological prophylaxis group, which was the same as our study [8]. In a meta-analysis of critically ill patients in 2022, the incidence of DVT was lower in the IPC group than in the no prophylaxis group, but there was no difference in the incidence of DVT between the pharmacological prophylaxis group or the combination of pharmacological prophylaxis and IPC [20]. In a meta-analysis of seven studies of gynecologic surgery patients, the incidence of DVT was lower in the IPC group than in the no-prophylaxis group but not in the pharmacological prophylaxis group, which was consistent with the results of this study [21]. Altogether, these results confirm that IPC is associated with a lower incidence of DVT than the no-prophylaxis group and no differences in incidence of DVT compared with the pharmacologic prophylaxis group.

The 2018 American Society of Hematology (ASH) guidelines recommend using pharmacological prophylaxis for VTE in acute or critically ill patients unless the effectiveness of IPC has been demonstrated to be superior [22]. However, as three meta-analyses, including this study, have shown no difference in DVT incidence between the IPC and pharmacologic prophylaxis groups, we believe efforts should be made to reflect this in clinical practice.

Six studies reported the incidence of PE in this meta-analysis: three in the pharmacological prophylaxis group and three in the combination of pharmacological prophylaxis and IPC, all of which were control groups. A meta-analysis of the overall effect size for PE rates reported in the six studies showed that the experimental group had a higher rate of PE than the control group. However, subgroup analyses to reduce heterogeneity showed no difference in PE rates between the experimental and control groups. A meta-analysis of PE rates in six studies of critically ill patients found similar PE rates in the experimental group receiving IPC and the control group receiving LMWH [20], and a meta-analysis of six studies of gynecologic surgery patients found no difference in PE rates between the experimental and control groups [21]. Antithrombotic medications are strongly recommended for patients at a high risk of DVT; however, anticoagulants, including heparin and LMWH, carry bleeding risks that can make it difficult for clinicians to use them promptly. Given that bleeding is a significant predictor of mortality, especially in surgical patients, and anticoagulation can increase blood loss and transfusion volumes [23], IPC should be strongly considered in patients at risk for bleeding. However, in a prospective cohort study by Lamontagne [24], non-leg venous thromboses were found in the deep and proximal veins in 2.2% of critically ill patients. Furthermore, a study by Park et al. [15] reported that non-leg venous thrombosis was more effective with pharmacologic prophylaxis. Therefore, it is necessary to consider pharmacologic prophylaxis or combination therapy with IPC in patients at high risk for PE.

However, unlike meta-analyses of critically ill patients or meta-analyses of gynecologic surgery patients, the three studies in this meta-analysis tested the difference in the incidence of PE after IPC and drug combination treatment, which may have resulted in different results because the treatment applied to the control group was different from that in previous meta-analysis studies. Additionally, previous studies have reported that various heparins used in the control group may affect the study results [21, 25]. Thus, conducting a comprehensive analysis of pharmacological prophylaxis applied to surgical patients in future studies is necessary.

Finally, to determine whether there was a difference in the incidence of bleeding between the experimental and control groups, we performed a meta-analysis of four studies that reported bleeding incidence as an outcome. Three of the four studies used pharmacological prophylaxis or IPC plus drug combination therapy in the control group, and the analysis showed that bleeding in the experimental group was significantly lower than in the control group.

The results of this study confirm previous findings [26] that IPC can prevent DVT by applying compression and decompression from the distal to the proximal part of the subject’s lower extremity. Furthermore, the fact that the application of IPC did not increase the incidence of DVT compared to pharmacological prophylaxis or IPC plus pharmacological prophylaxis and was associated with a lower number of bleeding events is of great relevance when considering that the patients in this study were hospitalized in a surgical ward after surgery.

In surgical patients, bleeding events and discontinuing thromboprophylaxis interventions can negatively affect clinical outcomes. Thromboprophylaxis should be based on the patient’s individualized bleeding [15]. Surgical patients are at increased risk of bleeding-related complications from invasive procedures compared to internal medicine patients, and based on the results of this meta-analysis, we believe that applying IPC in clinical practice to prevent DVT in surgical patients may be beneficial for patient safety. However, only 4 out of 16 studies reported adverse events related to bleeding; therefore, it is necessary to continue RCT to validate the findings of this study.

Despite these findings, this study has several limitations. First, there was clinical heterogeneity due to the different durations and methods of IPC use among the included studies. This may have influenced the occurrence of DVT. Therefore, caution should be exercised when interpreting these results. Second, the anticoagulants used for pharmacological prophylaxis in the control group differed in terms of drugs and dosage between the studies, and a few of the 16 studies included in this meta-analysis presented bleeding incidence as an outcome variable, which may cause skewness. Third, the high heterogeneity of surgery-related variables in the 16 studies precluded subgroup analysis. Finally, the 16 studies in this review used different methods to diagnose DVT. Thus, caution should be exercised when interpreting the results.

## Conclusions

This study conducted a systematic review and meta-analysis to provide a comprehensive and specific presentation of the effectiveness of IPC in preventing DVT and PE in patients hospitalized in surgical wards. The results of this study showed that IPC did not differ from pharmacotherapy in preventing DVT but was able to reduce the incidence of DVT compared with patients who did not receive any management for DVT prevention. However, the secondary outcome measure, bleeding, was significantly lower in the IPC group than in the pharmacological therapy group. As DVT prevention in clinical practice varies widely depending on the ward and patient situation, the results of this study can be used to improve nursing practice and provide consistent nursing interventions by applying IPC to prevent DVT in patients hospitalized in surgical wards. Since bleeding is a significant adverse event for patients admitted to the surgical ward after surgery, using the evidence identified in this study in the practice of preventing DVT in surgical patients may improve the quality of care provided to patients. Future studies should continue to confirm the effect of IPC on DVT incidence in surgical patients through well-designed RCTs. They should also be conducted to determine the effectiveness of IPC in different types of surgery.

## Supporting information

S1 Checklist PRISMA 2020 checklist. (DOCX)

S1 Appendix List of included studies. (DOCX)
