# Supplementary material for: Effects of intermittent pneumatic compression devices interventions to prevent deep vein thrombosis in surgical patients: A systematic review and meta-analysis of randomized controlled trials
Source: PLoS One. 2024 Jul 23;19(7):e0307602. doi: 10.1371/journal.pone.0307602 (PMC11265719; doi:10.1371/journal.pone.0307602)
Supplement: S1 Appendix — (DOCX) [file pone.0307602.s002.docx]

**Appendix: List of included studies**

A1. Domeij-Arverud E, Latifi A, Labruto F, Nilsson G, Ackermann PW. Can foot compression under a plaster cast prevent deep-vein thrombosis during lower limb immobilisation? Bone Joint J. 2013;95-B(9):1227-31. https://doi.org/10.1302/0301-620X.95B9.31162 PMID: 23997137

A2. Domeij-Arverud E, Labruto F, Latifi A, Nilsson G, Edman G, Ackermann PW. Intermittent pneumatic compression reduces the risk of deep vein thrombosis during post-operative lower limb immobilisation: a prospective randomised trial of acute ruptures of the Achilles tendon. Bone Joint J. 2015;97-B(5):675-80. https://doi.org/10.1302/0301-620X.97B5.34581 PMID: 25922463

A3. Coe NP, Collins RE, Klein LA, Bettmann MA, Skillman JJ, Shapiro RM, et al. Prevention of deep vein thrombosis in urological patients: a controlled, randomized trial of low-dose heparin and external pneumatic compression boots. Surgery. 1978;83(2):230-34. PMID: 622696

A4. Butson AR. Intermittent pneumatic calf compression for prevention of deep venous thrombosis in general abdominal surgery. Am J Surg. 1981;142(4):525-27. https://doi.org/10.1016/0002-9610(81)90391-3 PMID: 7025675

A5. Skillman JJ, Collins RE, Coe NP, Goldstein BS, Shapiro RM, Zervas NT, et al. Prevention of deep vein thrombosis in neurosurgical patients: a controlled, randomized trial of external pneumatic compression boots. Surgery. 1978;83(3):354-8. PMID: 628896.

A6. Wilson NV, Das SK, Kakkar VV, Maurice HD, Smibert JG, Thomas EM, et al. Thrombo-embolic prophylaxis in total knee replacement. Evaluation of the A-V Impulse System. J Bone Joint Surg Br. 1992;74(1):50-2. https://doi.org/10.1302/0301-620X.74B1.1732265 PMID: 1732265

A7. Wang JP, Lin YD, Wang L, Xu FG, Gao Y, Li CJ, et al. Effect of intermittent pneumatic compression on coagulation function and deep venous hemodynamics of lower limbs after rectal cancer resection. Zhonghua Wei Chang Wai Ke Za Zhi. 2013;16(8):739-43 PMID: 23980044

A8. Blanchard J, Meuwly JY, Leyvraz PF, Miron MJ, Bounameaux H, Hoffmeyer P, et al. Prevention of deep-vein thrombosis after total knee replacement. Randomised comparison between a low-molecular-weight heparin (nadroparin) and mechanical prophylaxis with a foot-pump system. J Bone Joint Surg Br. 1999;81(4):654-9. https://doi.org/10.1302/0301-620x.81b4.9464 PMID: 10463739

A9. Clarke-Pearson DL, Synan IS, Dodge R, Soper JT, Berchuck A, Coleman RE. A randomized trial of low-dose heparin and intermittent pneumatic calf compression for the prevention of deep venous thrombosis after gynecologic oncology surgery. Am J Obstet Gynecol. 1993;168(4):1146-53; discussion 1153-4. https://doi.org/10.1016/0002-9378(93)90360-u PMID: 8475960

A10. Maxwell GL, Synan I, Dodge R, Carroll B, Clarke-Pearson DL. Pneumatic compression versus low molecular weight heparin in gynecologic oncology surgery: a randomized trial. Obstet Gynecol. 2001;98(6):989-95. https://doi.org/10.1016/s0029-7844(01)01601-5 PMID: 11755543

A11. Nagata C, Tanabe H, Takakura S, Narui C, Saito M, Yanaihara N, et al. Randomized controlled trial of enoxaparin versus intermittent pneumatic compression for venous thromboembolism prevention in Japanese surgical patients with gynecologic malignancy. J Obstet Gynaecol Res. 2015;41(9):1440-8. https://doi.org/10.1111/jog.12740 PMID: 26111609

A12. Pitto RP, Hamer H, Heiss-Dunlop W, Kuehle J. Mechanical prophylaxis of deep-vein thrombosis after total hip replacement a randomised clinical trial. J Bone Joint Surg Br. 2004;86(5):639-42. https://doi.org/10.1302/0301-620x.86b5.14763 PMID: 15274256

A13. Stannard JP, Harris RM, Bucknell AL, Cossi A, Ward J, Arrington ED. Prophylaxis of deep venous thrombosis after total hip arthroplasty by using intermittent compression of the plantar venous plexus. Am J Orthop (Belle Mead NJ). 1996;25(2):127-34. PMID: 8640382

A14. Jung YJ, Seo HS, Park CH, Jeon HM, Kim JI, Yim HW, et al. Venous thromboembolism incidence and prophylaxis use after gastrectomy among Korean patients with gastric adenocarcinoma: The PROTECTOR randomized clinical trial. JAMA Surg. 2018;153(10):939-46. https://doi.org/ 10.1001/jamasurg.2018.2081 PMID: 30027281

A15. Song KY, Yoo HM, Kim EY, Kim JI, Yim HW, Jeon HM, et al. Optimal prophylactic method of venous thromboembolism for gastrectomy in Korean patients: an interim analysis of prospective randomized trial. Ann Surg Oncol. 2014;21(13):4232-38. https://doi.org/10.1245/s10434-014-3893-1 PMID: 25012265

A16. Kamachi H, Homma S, Kawamura H, Yoshida T, Ohno Y, Ichikawa N, et al. Intermittent pneumatic compression versus additional prophylaxis with enoxaparin for prevention of venous thromboembolism after laparoscopic surgery for gastric and colorectal malignancies: multicentre randomized clinical trial. BJS Open. 2020;4(5):804-10. https://doi.org/10.1002/bjs5.50323 PMID: 32700415
